# ﻿*Boreolimnus*, a new leafhopper genus from northern North America, with a review of *Cribrus* Oman (Hemiptera, Cicadellidae, Deltocephalinae)

**DOI:** 10.3897/zookeys.1217.126602

**Published:** 2024-11-07

**Authors:** Joel H. Kits

**Affiliations:** 1 Canadian National Collection of Insects, Arachnids, and Nematodes, Ottawa Research and Development Centre, Agriculture and Agri-Food Canada, Ottawa, Canada Canadian National Collection of Insects, Arachnids, and Nematodes Ottawa Canada

**Keywords:** COI barcodes, morphology, new genus, Paralimnini, phylogeny

## Abstract

The poorly known leafhopper species described as Deltocephalus (Laevicephalus) concinnus var. incisurus DeLong, 1926 previously had no accepted generic placement. It is here redescribed and placed in *Boreolimnus***gen. nov.** in the tribe Paralimnini, as *Boreolimnusincisurus* (DeLong) **comb. nov.***Cribrusmicmac* Hamilton, 1987 is a junior **syn. nov.** of *B.incisurus*. Due to historic confusion, the species currently placed in *Cribrus* Oman, 1949 were also reviewed. *Cribrusconcinnus* (Sanders & DeLong, 1917) is redescribed, and a lectotype is designated to clarify the application of the name. *Deltocephalusplagus* Ball & DeLong, 1926 and *Laevicephalusshingwauki* Beamer & Tuthill, 1934 are recognized as junior **syn. nov.** of *C.concinnus*, now the only recognized species in the genus.

## ﻿Introduction

The leafhopper taxon described by DeLong (1926) as Deltocephalus (Laevicephalus) concinnus var. incisurus has received little notice in the literature. DeLong described the taxon from a single specimen from Wisconsin. DeLong and Caldwell (1937) first treated *Laevicephalus* DeLong as a full genus, including this species as L.concinnusvar.incisurus. Beamer (1938) later treated it as a full species and described the male genitalia based on additional material from Manitoba. Oman (1949) listed the species as unplaced within a broadly defined Deltocephalini. Ross and Hamilton (1972) recognized *Latalushultus* Beirne as a junior synonym of *D.incisurus* and excluded the species from *Laevicephalus*, but they did not provide a new combination. It does not appear to have been mentioned in the literature since.

*Deltocephalusincisurus* belongs to the tribe Paralimnini as delimited by Zahniser and Dietrich (2013), based on the linear connective articulated with the aedeagus. This is a very diverse group of leafhoppers, with over 139 genera recognized (Zahniser and Dietrich 2013); 35 of these genera occur in the Nearctic region. There is currently no global treatment covering all genera in the tribe, but keys, descriptions, and illustrations in regional works (Oman 1949; Ribaut 1952; Ossiannilsson 1983; Anufriev and Emeljanov 1988; Emeljanov 1999; Li et al. 2011) suggest *D.incisurus* does not belong to any Nearctic or Palearctic genus as currently defined; thus, it is here placed in a new genus.

Although *D.incisurus* is clearly distinct from the Nearctic paralimnine genus *Cribrus* Oman based on male and female genitalia and wing venation, they share similar colour patterns and there has been previous confusion between the two. The holotype of *D.incisurus* is one of the two syntypes of *Cribrusconcinnus* (Sanders & DeLong), and examination of the holotype and only known specimen of *Cribrusmicmac* Hamilton showed that it is conspecific with *D.incisurus*. Thus, the other species currently placed in *Cribrus* were also reviewed to determine whether they are correctly placed and clarify their taxonomy.

While morphological evidence suggests that both genera examined here are distinct from other Paralimnini, molecular evidence was also examined as a further test of their status. The most comprehensive phylogeny of the Paralimnini is that of Cao et al. (2022), but neither of the taxa studied here were included. The only molecular data available for the two taxa is from the cytochrome oxidase I (COI) gene; hence, newly generated and previously published data for this gene were gathered from a number of Paralimnini in order to generate a phylogeny.

## ﻿Methods

### ﻿Depositories of types and other specimens examined are as follows

**CNC**Canadian National Collection of Insects, Arachnids, and Nematodes (Ottawa, Ontario, Canada)

**INHS**Illinois Natural History Survey (Champaign, Illinois, USA)

**OSUC**C.A. Triplehorn Insect Collection, The Ohio State University (Columbus, Ohio, USA)

**SEMC** Snow Entomological Museum Collection, University of Kansas (Lawrence, Kansas, USA)

**USNM**Smithsonian National Museum of Natural History (Washington, DC, USA)

Images were taken using a Leica M205C stereomicroscope with 1.6× objective (Leica Microsystems GmbH, Wetzlar, Germany), with infinity-corrected 5× or 10× objectives (Mitutoyo Corp., Kawasaki, Japan) mounted on a Canon R10 camera (Canon Inc., Tokyo, Japan) via a Thorlabs ITL200 tube lens (Thorlabs Inc., Newton, NJ, USA), or Nikon Eclipse E800 compound microscope with 10× or 20× objectives (Nikon Corp., Tokyo, Japan). Images were stacked using Zerene Stacker (Zerene Systems, Richland, WA, USA), edited using Adobe Photoshop CS6, and assembled into plates using Adobe Illustrator CS6 (Adobe Inc., San Jose, CA, USA). Morphological terminology follows Dietrich (2005).

Additional occurrence data for mapping *Cribrus* distribution were obtained from INHS (McElrath 2023) and from Jim Bess (pers. comm. 2024). All locality data with georeferences is in Suppl. material 2. Maps were created using QGIS 3.20.0 (QGIS.org).

COI sequences from CNC specimens were generated as described by Foottit et al. (2014) and Kits (2023). Additional sequences from genera not represented in the CNC dataset were downloaded from GenBank. A sequence from *Maiestasdorsalis* (Motschulsky), in the sister tribe Deltocephalini (Cao et al. 2022), was used to root the tree. Sequences were aligned using MAFFT 7.520 (Katoh and Standley 2013) and analysed using maximum likelihood in IQTREE 2.3 (Nguyen et al. 2015), with the model GTR+F+I+R5 selected by ModelFinder (Kalyaanamoorthy et al. 2017). Support values were calculated with 1000 rounds of ultrafast bootstrap (Hoang et al. 2018) and 1000 rounds of the Shimodaira-Hasegawa-like approximate likelihood-ratio test (SH-aLRT) (Guindon et al. 2010).

## ﻿Results

**Figure 1.**
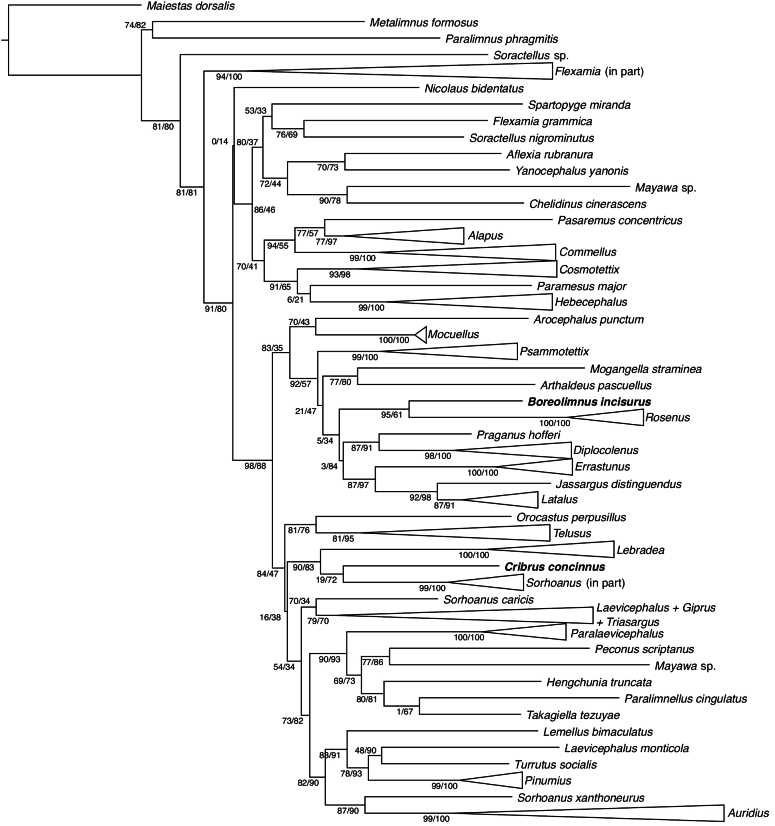
Maximum-likelihood tree of Paralimnini, based on 658 base pairs of cytochrome oxidase I. Selected clades collapsed to illustrate overall structure. Support values at nodes are bootstrap/SH-aLRT.

Sequences were obtained from 44 genera and 160 species of Paralimnini (Suppl. material 3). The phylogenetic analysis resolved most included genera as monophyletic with high support where multiple species were sampled (Fig. 1, Suppl. material 1). Exceptions include *Sorhoanus* Ribaut (polyphyletic with three distantly related clades), *Laevicephalus* (paraphyletic with respect to *Giprus* Oman and *Triasargus* Novikov & Anufriev, and with *L.monticola* (Gillette & Baker) distant from the main clade), and *Flexamia* DeLong (*F.grammica* (Ball) not recovered in the main clade).

Relationships between genera were generally only moderately or poorly resolved. *Boreolimnusincisurus* was resolved as sister to *Rosenus* Oman with fairly high support (95% bootstrap, 61 SH-aLRT). *Cribrusconcinnus* was resolved in a clade with *Lebradea* Remane and part of *Sorhoanus* with high support (90% bootstrap, 83 SH-aLRT), with a sister relationship to the latter weakly supported (19% bootstrap, 72 SH-aLRT).

### ﻿Taxonomic treatment

#### ﻿ Boreolimnus gen. nov.

Taxon classificationAnimaliaHemipteraCicadellidae

616EBECB-ABB6-5D99-80E4-874AD800F22A

https://zoobank.org/A2032F89-FA96-4946-87CA-77064621B4A3

##### Type species.

Deltocephalus (Laevicephalus) concinnus var. incisurus DeLong, 1926 (here designated)

##### Etymology.

The name is derived from the Greek βορέας (north) and λίμνη (marsh), describing the habitat of the type species. The gender is masculine.

##### Diagnosis.

Separated from other genera of Paralimnini by the following combination of characters: male subgenital plates with uniseriate macrosetae, plates as long as pygofer and tapering to a narrow rounded apex; pygofer with a process on postero-ventral margin, process nearly straight; segment X about as long as wide, broadly scerotized laterally and narrowly sclerotized dorsally; connective linear and elongate with arms fused in a stem which is about as broad as long (connective loop-shaped sensu Emeljanov (1999)); aedeagus broad and dorsoventrally flattened with subapical ventral gonopore and one pair of pre-apical processes; frontoclypeus and pronotum with longitudinal stripes; wings macropterous, fore wing with outer anteapical cell short and closed by distal fusion of veins R2+3 and R4+5 or absent.

##### Description.

**Figures 2–14.**
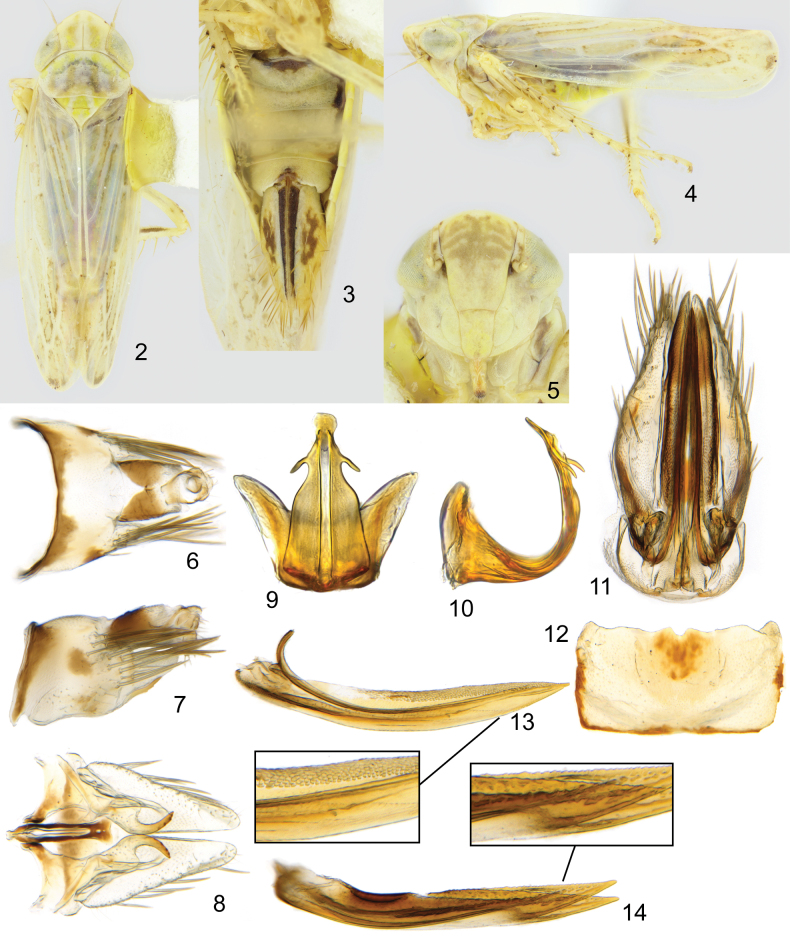
*Boreolimnusincisurus***2** dorsal habitus **3** lateral habitus **4** abdomen ventral, female **5** face **6** male pygofer, dorsal **7** male pygofer, lateral **8** male subgenital plate, styles, connective, dorsal **9** aedeagus, caudal **10** aedeagus, lateral **11** female genital capsule, ventral **12** female sternite VII, ventral **13** first valvifer, lateral, with enlargement **14** second valvifers, lateral, with enlargement.

Small leafhoppers with typical Paralimnini structure. Colour generally stramineous, head and pronotum with longitudinal stripes, wing with brown infuscation around cell borders (Figs 2–5).

Head with crown bluntly angled, medial length about 1.5× width between eyes (Fig. 2). Crown glabrous with fine striations on basal 2/3, distal 1/3 of crown and face shagreen. Lateral frontal sutures terminating lateral of ocelli, ocelli about 2× their own diameter from eye (Fig. 5). Mesal margin of eye notched. Anteclypeus with margins nearly straight, slightly tapered pre-apically. Lorum about 3/5 width of anteclypeus, well separated from genal margin. Antennae about as long as head width.

Pronotum slightly narrower than width of head across eyes, slightly longer than medial length of head. Fore femur with AM1 near ventral margin, row IC with a few fine setae, row AV consisting of a few, widely spaced, very short setae. Fore tibia with 1 AD and 4 PD macrosetae. Hind femur with 2+2+1 macrosetae. First hind tarsomere with two rows of plantar setae, four apical platellae between a pair of normal setae. Fore wing usually with three closed anteapical cells; outer anteapical cell short and closed by distal fusion of R2+3 and R4+5 or occasionally absent.

Male abdomen with apodemes on sternite II about twice as long as wide, apical half transparent, strongly curved dorsally. Pygofer about as long as wide, with a triangular distal lobe and a sclerotized process from posteroventral corner; with a patch of long macrosetae posterodorsally and shorter fine macrosetae scattered ventrally (Figs 6, 7). Segment X about as long as wide, heavily sclerotized laterally, the sclerotized portions narrowly connected posteriorly and separated by a V-shaped unsclerotized area medially. Valve parabolic. Subgenital plates as long as pygofer, subtriangular, with a narrowly rounded apex, bearing a single row of macrosetae (Fig. 8). Connective with arms nearly parallel, slightly bowed outwards towards anterior end and fused anteriorly, stem broadened apically, wider than arms and about as long as broad. Style apophysis with lateral lobe prominent, medial lobe with rounded teeth ventrally. Aedeagus dorsoventrally flattened with subapical ventral gonopore and one pair of pre-apical processes (Figs 9, 10).

Female pygofer with moderate length macrosetae (Fig. 11). Ovipositor not projecting beyond pygofer. Gonoplac without macrosetae. First valvula slightly concave; sculpture imbricate dorsally and strigate ventrally (Fig. 13). Second valvulae evenly tapered distally, with fine irregular dorsal teeth (Fig. 14).

##### Remarks.

*Boreolimnus* runs to *Latalus* in the keys of both Oman (1949) and Beirne (1956), but can be distinguished by several characters (alternative states in parentheses): outer anteapical cell reduced and closed by fusion of R2+3 and R4+5 (well developed and closed by crossvein s), connective narrow and nearly linear, with the posterior plate the widest part (connective broad and widest across the arms), aedeagus dorsoventrally flattened (aedeagus tubular, not flattened), frontoclypeus with longitudinal stripes (frontoclypeus with pale transverse markings separating darker areas). In Emeljanov (1999), it keys to couplet 300/307 but does not match either alternative well. In Ossiannilsson (1983) it keys best to *Lebradea*, from which is differs in the following characters: segment X about as long as wide (segment X about twice as long as wide), connective with arms connected posteriorly by a broad and long plate-like stem (connective with arms connected by a narrow bar-like stem posteriorly), stramineous with longitudinal stripes on frontoclypeus and pronotum and brown infuscation around wing cells (mostly bright yellow with black areas, no longitudinal stripes or infuscation on wing).

#### ﻿ Boreolimnus incisurus

Taxon classificationAnimaliaHemipteraCicadellidae

(DeLong) comb. nov.

F29845A8-124C-5F68-8A8C-B1C807AEEC3A

Figs 2–14 , 15–24

Deltocephalus (Laevicephalus) concinnus var. incisurus DeLong, 1926: 77
Laevicephalus concinnus var. incisurus (DeLong). Comb. DeLong and Caldwell 1937.
Laevicephalus incisurus (DeLong, 1926). Rev. stat. Beamer 1938.
= Latalushultus Beirne, 1954: 123. Syn. Ross and Hamilton 1972.
= Cribrusmicmac Hamilton in Hamilton and Langor 1987: 669. New synonym.

##### Description.

Males 3.1–3.4 mm. Females 3.2–3.6 mm.

Colour mostly pale straw to light yellow, with two longitudinal stripes on crown and four longitudinal stripes on pronotum in a deeper yellow colour usually apparent. Palest specimens with dark colour restricted to basal tergites and spots at bases of leg macrosetae. Darker specimens may have light to dark brown markings medially on frontoclypeus (interrupted laterally by pale horizontal lines), in antennal pits, on anepisternum, medially on abdominal tergites, on base of sternite II and laterally on all sternites, and on pygofer. Fore wing milky white with brown infuscation around border of some cells.

Male pygofer process short, originating on postero-ventral margin and extending slightly dorsally. Process typically with two small teeth on ventral margin. Subgenital plates bearing a single row of approximately eight macrosetae laterally. Style with lateral lobe of apophysis quadrately rounded, medial lobe of apophysis sickle-shaped, with four or five widely spaced, rounded teeth ventrally. Aedeagus in lateral view dorsoventrally flattened, strongly curved anterodorsally, extending slightly dorsally of atrium. Atrium in posterior view with deep and broad dorsal excavation; shaft in posterior view broad, narrowing preapically, with a single pair of lateral processes just before apex, terminating in a round plate above gonopore.

Female sternite VII rectangular, posterior margin with slight, rounded projections medially and laterally and gently convex in between, medial projection with a small emargination surrounded by a dark area (Fig. 12). Gonoplac mostly dark. Base of first valvula in ventral view truncate (Fig. 11).

##### Material examined.

***Holotype*** of *Deltocephalusincisurus* DeLong. USA • ♀; Wisconsin, Ladysmith; 9 Aug. 1916; D.M. DeLong leg.; OSUC, OSUC 870278.

***Holotype*** of *Latalushultus* Beirne. Canada • ♂; Manitoba, Birch River; 13 Aug. 1930; R.H. Handford leg.; CNC, CNC1197446.

***Holotype*** of *Cribrusmicmac* Hamilton. Canada • ♀; Nova Scotia, Cape Breton Highlands National Park, Paquet’s Lake; 27 Aug. 1983; M. Sharkey leg.; CNC, CNC#HEM403381.

##### Other material.

Canada – **Alberta** • 1 ♂; Beaverlodge; 1 Aug. 1961; A.R. Brooks leg.; CNC • 1 ♀; Grande Prairie; 25 Jul. 1961; A.R. Brooks leg.; CNC • 1 ♂; High Prairie; 16 Jul. 1961; A.R. Brooks leg.; CNC • 20 ♂, 12 ♀, 1 (no abdomen); same collection data as previous; 17 Jul. 1961; CNC • 18 ♂, 16 ♀, 2 (intersex); same collection data as previous; 22 Jul. 1961; CNC • 1 ♂; same collection data as previous; 25 Jul. 1961; CNC • 1 ♀; same collection data as previous; 26 Jul. 1961; CNC • 3 ♂, 1 ♀; Peace River; 12 Jul. 1961; A.R. Brooks leg.; CNC • 1 ♂, 7 ♀, 1 (intersex); Valleyview; 10 Aug. 1961; A.R. Brooks leg; CNC. – **New Brunswick** • 1; Kouchibouguac National Park; 16 Aug. 1977; S.J. Miller leg.; CNC. – **Ontario** • 1 ♀; 10 mi E Nipigon; 12 Aug. 1975; K.G.A. Hamilton leg.; CNC • 1 ♀; 4 mi S Beardmore; 12 Aug. 1975; K.G.A. Hamilton leg.; from *Calamagrostiscanadensis*; CNC • 28 (unmounted specimens in a capsule); Sault Sainte Marie; 10 Aug. 1975; K.G.A. Hamilton leg.; from *Carex* sp.; CNC. – **Saskatchewan** • 4 ♀; Candle Lake; 19 Aug. 1959; A. & J. Brooks leg.; CNC. – **Manitoba** • 1 ♀; The Pas; 30 Aug. 1959; A. & J. Brooks leg.; CNC.

##### Remarks.

**Figures 15–24.**
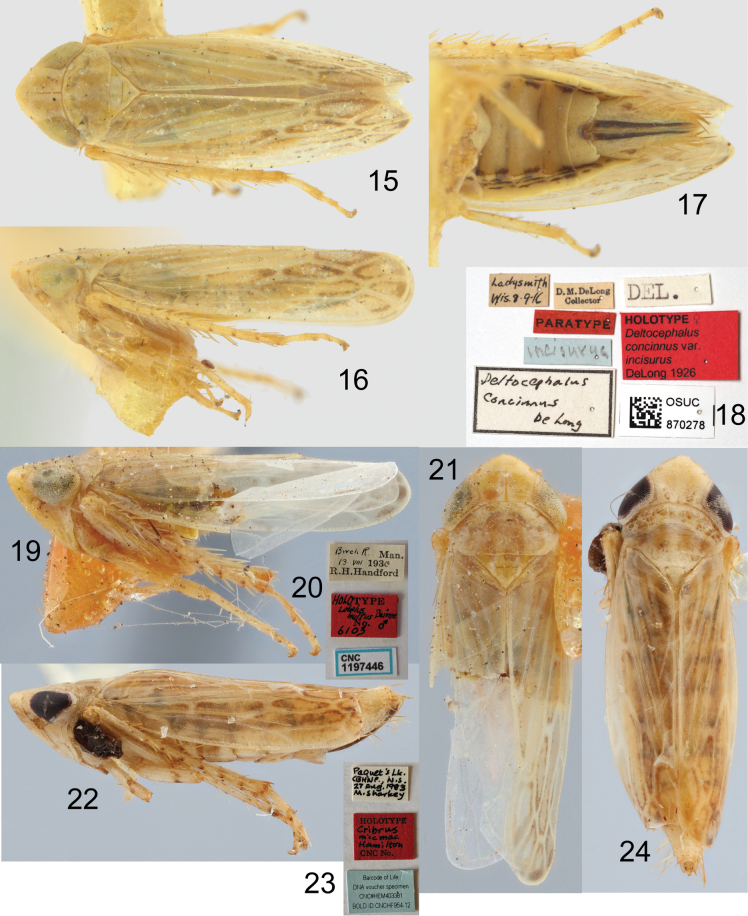
*Boreolimnusincisurus* and synonyms, primary types **15–18**Deltocephalusconcinnusvar.incisurus DeLong, holotype **15** dorsal habitus **16** lateral habitus **17** abdomen, ventral **18** labels **19–21***Latalushultus* Beirne, holotype **19** lateral habitus **20** labels **21** dorsal habitus **22–24***Cribrusmicmac* Hamilton, holotype **22** lateral habitus **23** labels **24** dorsal habitus.

The holotype of *Deltocephalusincisurus* (Figs 15–18) was not previously labelled as such in the OSUC collection. The red “paratype” label and blue “incisurus” label were both probably added by later workers. However, it seems clear this is the holotype, as it matches the locality data, description, and illustrations in DeLong (1926). This specimen is also presumably one of the two syntypes of *Deltocephalusconcinnus* Sanders & DeLong, based on the labels which match the data in the original description and the fact that no other potential syntypes could be located in DeLong’s collection (L. Musetti pers. comm. 2022). As I am designating the other syntype as lectotype of *D.concinnus* (see below), this specimen becomes a paralectotype of the latter species.

The holotype of *Latalushultus* (Figs 19–21) has been dissected, and matches other males of this species. The original description (Beirne 1954a) did not include collection details for the holotype; these were provided in an erratum (Beirne 1954b). The latter also mentions a paratype which could not be located in the CNC; its whereabouts are unknown.

The holotype of *Cribrusmicmac* (Figs 22–24) has the fore wings reaching about the middle of the genital segment. The hind wing length is difficult to determine precisely as the wings are greasy and stuck together, but they appear to be about as long as the fore wings. In all other examined females, both wings exceed the apex of the genital segment although there is some variation in how far they exceed the apex. The holotype appears to have been parasitized, with a dark mass resembling a dryinid larval sac projecting between the first and second thoracic segments; this could have caused abnormal development of the wings. The seventh sternite also has a shallower medial emargination compared to other females, but again this may represent abnormal development due to parasitization. Otherwise, the holotype matches other examined females in structure and colour, and the small COI fragment available for the specimen (GenBank accession PP719690, 137 bp) is 100% identical to the sequence from *B.incisurus* included in the phylogenetic analysis.

Females of this species can be separated from *Cribrus* and other Nearctic Paralimnini with longitudinal stripes on the head and pronotum based on the distinctly infuscated cell borders of the fore wing, reduction of the outer anteapical cell, sternite VII with slightly projecting posterior corners and a small darkened emargination medially, and dark gonoplacs.

##### Distribution.

**Figure 25.**
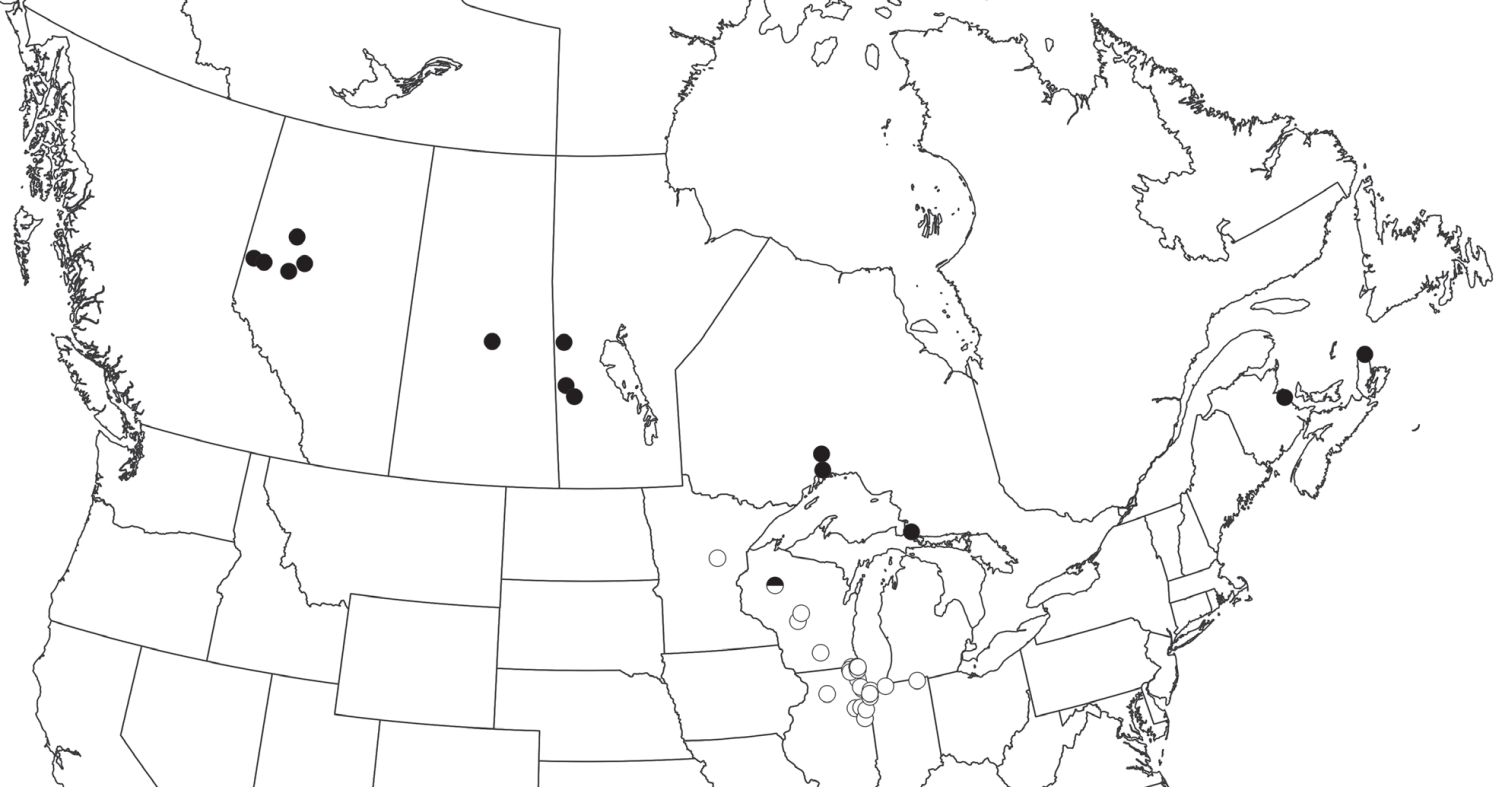
Map of localities for *Boreolimnusincisurus* (black dots) and *Cribrusconcinnus* (white dots). The half-filled dot is Ladysmith, Wisconsin, type locality for both species and the only known co-occurrence.

Recorded from Alberta to Nova Scotia, south to Wisconsin (Fig. 25). Locations largely fall within the southern boreal forest or transition zones.

##### Host plants.

Associated with graminoids in northern wetlands, although the specific host is unclear. The type specimen was collected from “grasses on the margin of a tamarack bog” (DeLong 1926). Beamer’s collection from Cowan, MB was probably collected from grasses along the margin of a lake (based on Beamer’s collection notes for this locality, as quoted by Whitcomb and Hicks 1988: 323). One specimen from near Beardmore, ON was collected from *Calamagrostiscanadensis*, while a large series from Sault Ste. Marie, ON was collected from *Carex* sp. All three Ontario localities were wetlands with *Calamagrostiscanadensis* as a dominant species (K.G.A. Hamilton field notes, unpublished) and this common wetland grass is a potential candidate for the host plant, but further fieldwork is needed.

#### ﻿ Cribrus

Taxon classificationAnimaliaHemipteraCicadellidae

Oman, 1949

84ECCCF3-5A0F-5166-B8A2-4B7751726302

##### Type species.

*Laevicephalusshingwauki* Beamer & Tuthill, 1934, by original designation (Oman 1949: 166).

##### Diagnosis.

Separated from other genera of Paralimnini by the following combination of characters: male subgenital plates with uniseriate macrosetae, plates truncate and shorter than pygofer; pygofer without processes; pygofer with a prominent pair of dorsal spots; connective linear with posterior stem about as long as wide (connective loop-shaped sensu Emeljanov (1999)); aedeagus with swollen atrium, short shaft with apical gonopore and one pair of apical processes; frontoclypeus and pronotum with longitudinal stripes; wings usually brachypterous, fore wing with three closed anteapical cells.

##### Description.

**Figures 26–40.**
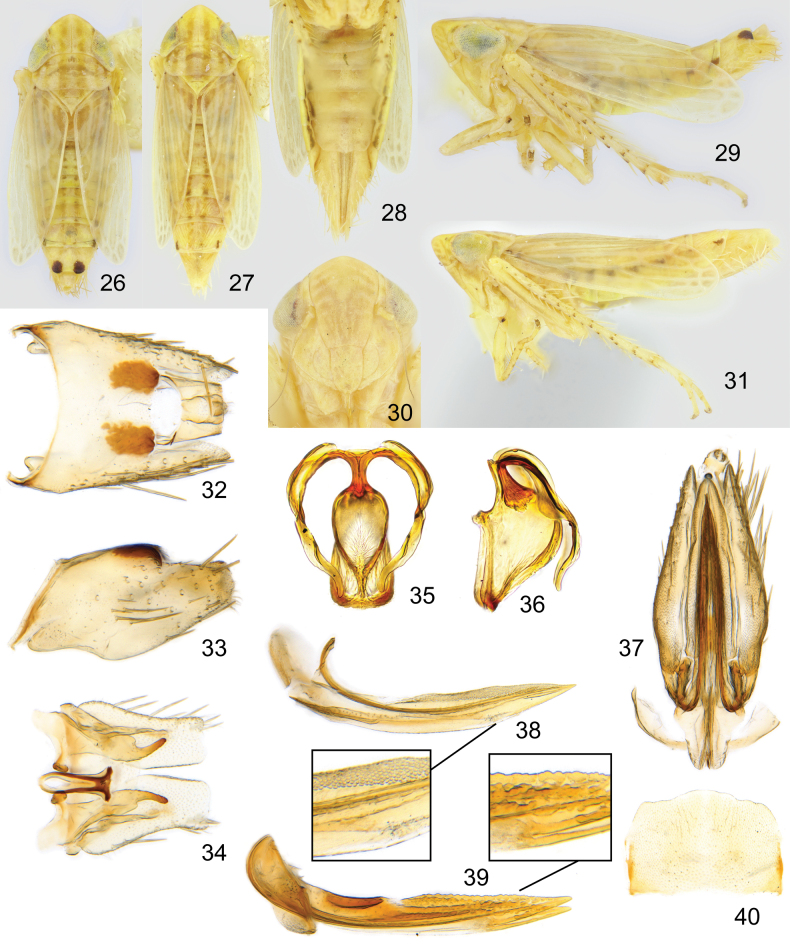
*Cribrusconcinnus***26** dorsal habitus, male **27** dorsal habitus, female **28** abdomen ventral, female **29** lateral habitus, male **30** face **31** lateral habitus, female **32** male pygofer, dorsal **33** male pygofer, lateral **34** male subgenital plate, styles, connective, dorsal 35 aedeagus, caudal **36** aedeagus, lateral **37** female genital capsule, ventral **38** first valvifer, lateral, with enlargement **39** second valvifers, lateral, with enlargement **40** female sternite VII, ventral.

Small leafhoppers with typical Paralimnini structure. Colour generally stramineous, head and pronotum with longitudinal stripes, wing with indistinct brown infuscation around cell borders (Figs 26–31).

Head with crown bluntly angled, medial length about equal to width between eyes (Figs 26, 27). Crown glabrous at base, margin and face shagreen. Lateral frontal sutures terminating just ventral of ocelli, ocelli about their own diameter distant from eye (Fig. 30). Mesal margin of eye notched. Anteclypeus with margins slightly convex, distal third distinctly tapered. Lorum about half width of anteclypeus, well separated from genal margin. Antennae about as long as head width.

Pronotum slightly narrower than width of head across eyes, about as long as medial length of head. Fore femur with AM1 near ventral margin, row IC with a few fine setae, row AV consisting of a few, widely spaced, very short setae. Fore tibia with 1 AD and 4 PD macrosetae. Hind femur with 2+2+1 macrosetae. First hind tarsomere with two rows of plantar setae, four apical platellae between a pair of longer normal setae. Fore wing with three closed anteapical cells, although venation may be distorted due to brachyptery.

Male abdomen with apodemes on sternite II poorly developed, shorter than width. Pygofer about twice as long as wide, with a patch of long macrosetae posterodorsally and a few small macrosetae scattered ventrally (Figs 32, 33). Pygofer dorsally with a pair of heavily sclerotized spots basal to segment X. Segment X about as long as wide, completely sclerotized dorsally and laterally. Valve parabolic. Subgenital plates truncate, shorter than pygofer, bearing a single row of macrosetae laterally (Fig. 34). Connective with arms fused anteriorly, tapered towards posterior end, stem abruptly broadened apically, wider than arms. Style apophysis with lateral lobe weakly developed, medial lobe with corrugated sculpture but no distinct teeth. Aedeagus with swollen atrium, shaft very short with apical gonopore, with one pair of apical processes (Figs 35, 36).

Female pygofer with moderate length macrosetae (Fig. 37). Ovipositor not projecting beyond pygofer. Gonoplac without macrosetae. First valvula slightly concave; sculpture imbricate dorsally and strigate ventrally (Fig. 38). Second valvulae evenly tapered distally, with rounded teeth decreasing in size distally (Fig. 39).

#### ﻿ Cribrus concinnus

Taxon classificationAnimaliaHemipteraCicadellidae

(Sanders & DeLong)

C9683B92-9036-5B81-A695-10052B89DBF0

Figs 26–40 , 41–43 , 44–47

Deltocephalus concinnus Sanders & DeLong 1917: 86.
Laevicephalus concinnus (Sanders & DeLong): Comb. Beamer and Tuthill 1934.
Cribrus concinnus (Sanders & DeLong): Comb. Ross and Hamilton 1972.
= Deltocephalusplagus Ball & DeLong, 1926: 241. New synonym.
= Laevicephalusshingwauki Beamer & Tuthill, 1934: 19. New synonym.

##### Description.

Males 2.5–2.8 mm. Females 3.3–3.6 mm.

Colour mostly light yellow, with two light brown longitudinal stripes on crown and four longitudinal stripes on pronotum. Legs with dark spots at bases of macrosetae. Abdominal tergites with four brown to black longitudinal stripes usually apparent. Abdominal sternites may have lateral brown markings. Fore wing pale brown with indistinct darker brown infuscation around border of cells. Wing length variable in females, from fully macropterous to brachypterous with fore wing reaching apex of tergite VI and hind wing reaching apex of tergite II. Males brachypterous with fore wing reaching base to midpoint of pygofer and hind wing reaching apex of tergite II to III.

Subgenital plates bearing a single row of approximately seven macrosetae laterally. Style with medial lobe of apophysis finger-shaped. Aedeagus with long apical processes curving toward base, sculptured with complex ridges.

Female sternite VII rectangular, posterior corners rounded, posterior margin straight to moderately convex, may have slight projections medially and laterally (Fig. 40). Gonoplac pale. Base of first valvula in ventral view elongate (Fig. 37).

##### Material examined.

***Lectotype*** of *Deltocephalusconcinnus* Sanders & DeLong (here designated). USA • ♀; Wisconsin, Ladysmith; 9 Aug. 1916; D.M. DeLong leg.; OSUC, OSUC 0171752.

***Holotype*** of *Deltocephalusplagus* Ball & DeLong. USA • ♀ (specimen missing from point, not examined); Wisconsin, Madison; 21 Sep. 1917; E.D. Ball leg.; USNM.

***Holotype*** of *Laevicephalusshingwauki* Beamer & Tuthill. USA • ♂ (apparently lost, not examined); Minnesota, Aitkin; 25 Aug. 1933; P.B. Lawson leg.; SEMC.

##### Other material.

USA – **Illinois** • 1 ♂, 3 ♀; 3 mi W Kankakee; 25 Aug. 1980; K.G.A. Hamilton leg.; CNC • 24 ♀; Fox Lake; 26 Jun. 1935; DeLong & Ross leg.; INHS • 2 ♂, 1 ♀; Fox Lake; 6 Aug. 1935; DeLong & Ross leg.; INHS • 3 ♀; Fox Lake; 26 Jun. 1936; Frison & DeLong leg.; INHS • 8 ♂, 7 ♀, 2 nymphs, approximately 20 unmounted specimens in a capsule; Iroquois Co., 7 mi NE Beaverville; 25 Sep. 1962; Ross & Ross leg.; from *Calamagrostiscanadensis*; GL 177; CNC • 1 ♀; Zion; 16 Jun. 1954; Sanderson & Moore leg.; CNC. – **Wisconsin** • 1 ♂; Juneau Co., 6 mi NE Mather; 17 Jul. 1963; Smith & Stannard leg.; GL 654; CNC • 8 ♀; Wood Co.; 16 Jul. 1963; Stannard & Smith leg.; GL 666; CNC.

##### Remarks.

**Figures 41–43.**
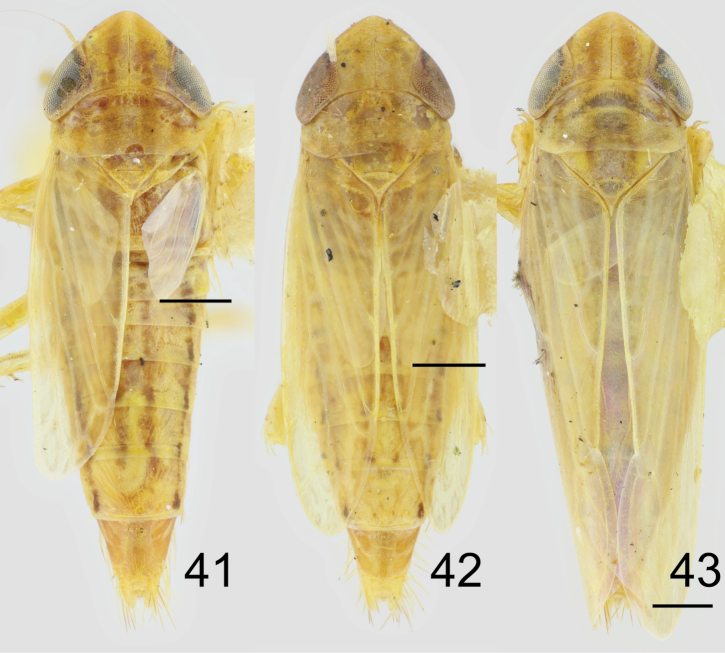
*Cribrusconcinnus*, dorsal habitus. Females collected at Fox Lake, Illinois, 30 June 1935. Horizontal lines mark apex of hind wing **41** shorter-winged brachypter, right forewing missing **42** longer-winged brachypter **43** macropter.

All males examined are brachypterous and clearly belong to a single species with distinctive genitalia. Females differing in wing length were previously treated as distinct species (e.g. DeLong 1948), but other structural features and colour pattern are consistent among specimens with different length wings. The examined series from Fox Lake, Illinois, collected on 30 June 1935, includes 23 brachypterous females with fore wing reaching the apex of tergites VI to VII and hind wing reaching the apex of tergites II to IV (all identified by D. DeLong as “*Laevicephalusshingwauki*”) (Figs 41, 42) and one macropterous female with fore and hind wings both exceeding the tip of the abdomen (identified by D. DeLong as “*Laevicephalusconcinnus*”) (Fig. 43). The specimens are otherwise inseparable, and the best explanation for the wing length variation among females is the presence of a rare macropterous morph within a single species. Synonymies in the genus are complicated by the apparent loss of the type material of *C.plagus* and *C.shingwauki*, but the available evidence suggests both be treated as junior synonyms of *C.concinnus*. With the synonymies proposed here, *Cribrus* becomes a monotypic genus including only *C.concinnus*.

**Figures 44–47.**
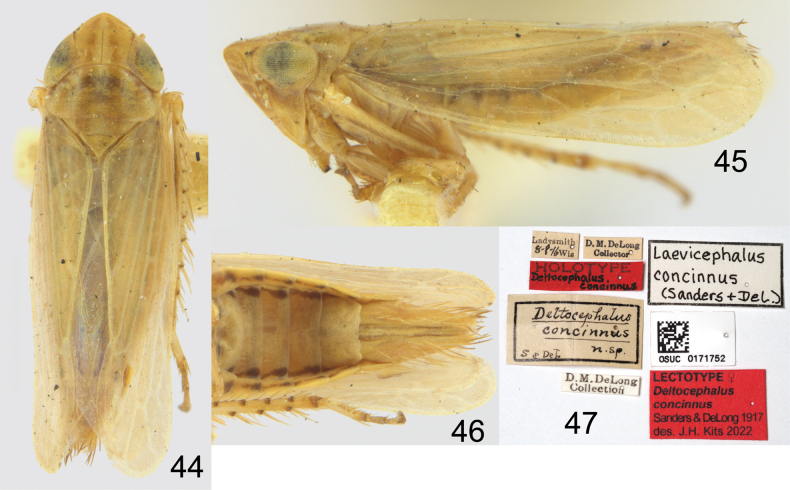
*Deltocephalusconcinnus* Sanders & DeLong, lectotype **44** dorsal habitus **45** lateral habitus **46** abdomen, ventral **47** labels.

Sanders and DeLong (1917) described *D.concinnus* from two female syntypes from the same locality. One of these was apparently later designated as the holotype of D.concinnusvar.incisurus as discussed above. There is no published lectotype designation for *D.concinnus*, and so I here designate the other syntype (Figs 44–47) as lectotype to stabilize the application of the name. This appears to be the specimen illustrated under this name by Sanders and DeLong (1917) and DeLong (1926). The specimen was labelled as “holotype” in DeLong’s collection in OSUC. However, this label was probably added later by another worker (L. Musetti pers. comm. 2022) and is incorrect, as no holotype was originally designated. The lectotype is a macropterous specimen, but it is otherwise indistinguishable from other female specimens of the species.

The holotype of *D.plagus* is missing from the point, with only a leg remaining (S. McKamey pers. comm. 2022). The original description and illustrations are both good matches to brachypterous females of this species. Oman (1949) suggested *D.plagus* was probably a synonym of *C.shingwauki*, although he did not formally synonymize them.

The holotype and a male paratype of *Laevicephalusshingwauki* are stated in the original description to be deposited in the SEMC, but they can not be located there now (R. Osborn pers. comm. 2024). Beamer and Tuthill (1934) separated their species from *C.concinnus* based on the smaller size, shorter wings, and abdominal colouration, although they speculated it might actually be the male of the former. Ross and Hamilton (1972) later also suggested that *C.concinnus* was “close to if not the same species as *C.shingwauki*.” Although the internal genitalia of the type series were not described, the description of the external characters and illustration of the external genitalia are a clear match to the present concept. The differences in size and abdominal colouration mentioned by Beamer and Tuthill (1934) both represent sexual dimorphism within the species.

Females of this species can be separated from *Boreolimnus* and other Nearctic Paralimnini with longitudinal stripes on the head and pronotum based on the longitudinal dorsal stripes on the abdomen, outer anteapical cell well developed and closed by crossvein s, sternite VII entirely pale with rounded posterior corners and without medial emargination, and pale gonoplacs.

##### Distribution.

Found in the midwestern United States (Minnesota to Indiana), around the eastern margin of the tallgrass prairie region (Fig. 25).

##### Host plants.

Associated with *Calamagrostis*, usually in mesic to wet prairie or wetlands (DeLong 1948; Panzer et al. 2003; J. Bess pers. comm.; examined specimens).

## ﻿Discussion

Although the Nearctic fauna of Paralimnini is fairly well known as a whole, many taxa have not yet been included in modern revisionary studies. In addition to various undescribed or unrecognized species, some currently recognized species are inadequately characterized and require more research to resolve their status. The new genus and new synonymies here resolve the status of a few of these obscure nominal species. Continued taxonomic research on other little-known taxa of Nearctic Paralimnini is needed to further advance the taxonomy of this diverse group of leafhoppers.

The phylogenetic analysis also points to potential issues in generic classification of the Paralimnini, with a few well-sampled genera recovered as non-monophyletic. However, the single gene used is insufficient for robust recovery of deeper, mostly intrageneric relationships, with low support values for most nodes near the base of the tree. Revisions to the generic classification should not be based on these results alone, but further analysis with multiple genes and dense taxon sampling should be prioritized.

## Supplementary Material

XML Treatment for Boreolimnus

XML Treatment for Boreolimnus incisurus

XML Treatment for Cribrus

XML Treatment for Cribrus concinnus

## ﻿Additional information

### Author ORCIDs

Joel H. Kits https://orcid.org/0000-0003-2685-0567

### Data availability

All of the data that support the findings of this study are available in the main text or Supplementary Information.

## Supplementary materials

Supplementary material 1 Maximum likelihood tree of Paralimnini, based on 658 base pairs of cytochrome oxidase I Data type tif Explanation note Support values at nodes are bootstrap/SH-aLRT. https://binary.pensoft.net/file/1169806This dataset is made available under the Open Database License (http://opendatacommons.org/licenses/odbl/1.0/). The Open Database License (ODbL) is a license agreement intended to allow users to freely share, modify, and use this Dataset while maintaining this same freedom for others, provided that the original source and author(s) are credited.Joel H. Kits

Supplementary material 2 Georeferenced localities for *Boreolimnusincisurus* and *Cribrusconcinnus* Data type csv https://binary.pensoft.net/file/1169807This dataset is made available under the Open Database License (http://opendatacommons.org/licenses/odbl/1.0/). The Open Database License (ODbL) is a license agreement intended to allow users to freely share, modify, and use this Dataset while maintaining this same freedom for others, provided that the original source and author(s) are credited.Joel H. Kits

Supplementary material 3 Data for COI sequences used in phylogenetic analysis Data type csv https://binary.pensoft.net/file/1169808This dataset is made available under the Open Database License (http://opendatacommons.org/licenses/odbl/1.0/). The Open Database License (ODbL) is a license agreement intended to allow users to freely share, modify, and use this Dataset while maintaining this same freedom for others, provided that the original source and author(s) are credited.Joel H. Kits

## References

[B1] Anufriev GA Emeljanov AF (1988) Suborder Cicadinea (Auchenorrhyncha). In: Lehr PA (Ed.) Keys to the Insects of the Far East of the USSR, Vol.2. Nauka, Leningrad, 12–495.

[B2] Ball ED DeLong DM (1926) Three new species of *Deltocephalus*. Journal of the New York Entomological Society 34: 241–242. https://www.jstor.org/stable/25004139

[B3] Beamer RH (1938) Miscellaneous leafhoppers with descriptions of five new species (Homoptera, Cicadellidae). The Canadian Entomologist 70: 224–230. 10.4039/Ent70224-11

[B4] Beamer RH Tuthill LD (1934) Some new species and a new genus of deltocephaloid leafhoppers (Homoptera, Cicadellidae). Journal of the Kansas Entomological Society 7: 1–24. http://www.jstor.org/stable/25081372

[B5] Beirne BP (1954a) Canadian species of *Latalus* (Homoptera: Cicadellidae). The Canadian Entomologist 86: 123–127. 10.4039/Ent86123-3

[B6] Beirne BP (1954b) Errata. The Canadian Entomologist 86: 192. 10.4039/Ent86192-4

[B7] Beirne BP (1956) Leafhoppers (Homoptera: Cicadellidae) of Canada and Alaska. Memoirs of the Entomological Society of Canada 88: 5–180. 10.4039/entm8802fv

[B8] Cao Y Dietrich CH Zahniser JN Dmitriev DA (2022) Dense sampling of taxa and characters improves phylogenetic resolution among deltocephaline leafhoppers (Hemiptera: Cicadellidae: Deltocephalinae). Systematic Entomology 2022: 1–15. 10.1111/syen.12540

[B9] DeLong DM (1926) A monographic study of the North American species of the genus *Deltocephalus*. The Ohio State University Studies 2(13), Contributions in Zoology and Entomology 3: 1–129.

[B10] DeLong DM (1948) The leafhoppers, or Cicadellidae, of Illinois (Eurymelinae–Balcluthinae). Bulletin of the Illinois Natural History Survey 24: 97–376. 10.21900/j.inhs.v24.196

[B11] DeLong DM Caldwell JS (1937) Check List of the Cicadellidae (Homoptera) of America, North of Mexico. The Ohio State University, Columbus, 93 pp.

[B12] Dietrich CH (2005) Keys to the families of Cicadomorpha and subfamilies and tribes of Cicadellidae (Hemiptera: Auchenorrhyncha). Florida Entomologist 88: 502–517. 10.1653/0015-4040(2005)88[502:KTTFOC]2.0.CO;2

[B13] Emeljanov AF (1999) A key to genera of the subfamily Deltocephalinae s.l. (Homoptera, Cicadellidae) from Kazakhstan, Middle Asia, and Mongolia with description of new genera and subgenera. Entomological Review 79: 547–562.

[B14] Foottit RG Maw E Hebert PDN (2014) DNA barcodes for Nearctic Auchenorrhyncha (Insecta: Hemiptera). PLoS ONE 9: e101385. 10.1371/journal.pone.0101385 PMC4087040 25004106

[B15] Guindon S Dufayard J-F Lefort V Anisimova M Hordijk W Gascuel O (2010) New algorithms and methods to estimate maximum-likelihood phylogenies: assessing the performance of PhyML 3.0. Systematic Biology 59: 307–321. 10.1093/sysbio/syq010 20525638

[B16] Hamilton KGA Langor DW (1987) Leafhopper fauna of Newfoundland and Cape Breton Islands (Rhynchota: Homoptera: Cicadellidae). The Canadian Entomologist 119: 663–95. 10.4039/Ent119663-7

[B17] Hoang DT Chernomor O von Haeseler A Minh BQ Vinh LS (2018) UFBoot2: improving the ultrafast bootstrap approximation. Molecular Biology and Evolution 35: 518–522. 10.1093/molbev/msx281 29077904 PMC5850222

[B18] Kalyaanamoorthy S Minh BQ Wong TKF von Haeseler A Jermiin LS (2017) ModelFinder: fast model selection for accurate phylogenetic estimates. Nature Methods 14: 587–589. 10.1038/nmeth.4285 28481363 PMC5453245

[B19] Katoh K Standley DM (2013) MAFFT multiple sequence alignment software version 7: improvements in performance and usability. Molecular Biology and Evolution 30: 772–780. 10.1093/molbev/mst010 23329690 PMC3603318

[B20] Kits JH (2023) The genus *Errastunus* in the Nearctic region (Hemiptera, Cicadellidae, Deltocephalinae). ZooKeys 1178: 143–164. 10.3897/zookeys.1178.105566 37711497 PMC10498272

[B21] Li Z Dai R Xing J (2011) Deltocephalinae from China (Hemiptera: Cicadellidae). Popular Science Press, Beijing, 336 pp.

[B22] McElrath T (2023) . Illinois Natural History Survey Insect Collection. Illinois Natural History Survey. Occurrence dataset. 10.15468/eol0pe [accessed via GBIF.org on 2024-04-23]

[B23] Nguyen L-T Schmidt HA von Haeseler A Minh BQ (2015) IQ-TREE: a fast and effective stochastic algorithm for estimating maximum likelihood phylogenies. Molecular Biology and Evolution 32: 268–274. 10.1093/molbev/msu300 25371430 PMC4271533

[B24] Oman PW (1949) The Nearctic Leafhoppers (Homoptera: Cicadellidae): a Generic Classification and Check List. Entomological Society of Washington, Washington, DC, 153 pp.

[B25] Ossiannilsson F (1983) The Auchenorrhyncha (Homoptera) of Fennoscandia and Denmark. Part 3: the Family Cicadellidae: Deltocephalinae, catalogue, literature, and index. Scandinavian Science Press, Copenhagen, 979 pp. 10.1163/9789004273320

[B26] Panzer R Derkovitz G Gnaedinger K (2003) A survey of the leafhoppers, planthoppers, froghoppers, grasshoppers, butterflies, and moths of the Green River State Wildlife Area, Lee County, Illinois. Illinois Department of Natural Resources unpublished report, 33 pp. https://dnr.illinois.gov/content/dam/soi/en/web/dnr/grants/documents/wpfgrantreports/2001l08w.pdf

[B27] Ribaut H (1952) Homoptères Auchénorhynques. II (Jassidae). In: Faune de France, Vol. 57. Paul Lechevalier, Paris, 474 pp.

[B28] Ross HH Hamilton KGA (1972) A review of the North American leafhopper genus *Laevicephalus* (Hemiptera: Cicadellidae). Annals of the Entomological Society of America 65: 929–942. 10.1093/aesa/65.4.929

[B29] Sanders JG DeLong DM (1917) The Cicadellidae (Jassoidea–Fam. Homoptera) of Wisconsin, with description of new species. Annals of the Entomological Society of America 10: 79–97. 10.1093/aesa/10.1.79

[B30] Whitcomb RF Hicks AL (1988) Genus Flexamia: new species, phylogeny, and ecology. Great Basin Naturalist Memoirs 12: 224–323. 10.5962/bhl.part.10987

[B31] Zahniser JN Dietrich CH (2013) A review of the tribes of Deltocephalinae (Hemiptera: Auchenorrhyncha: Cicadellidae). European Journal of Taxonomy 45: 1–211. 10.5852/ejt.2013.45

