# Supplementary figures and images for: ﻿Boreolimnus, a new leafhopper genus from northern North America, with a review of Cribrus Oman (Hemiptera, Cicadellidae, Deltocephalinae)
Source: Zookeys. 2024 Nov 7;1217:273–90. doi: 10.3897/zookeys.1217.126602 (PMC11565182; doi:10.3897/zookeys.1217.126602)

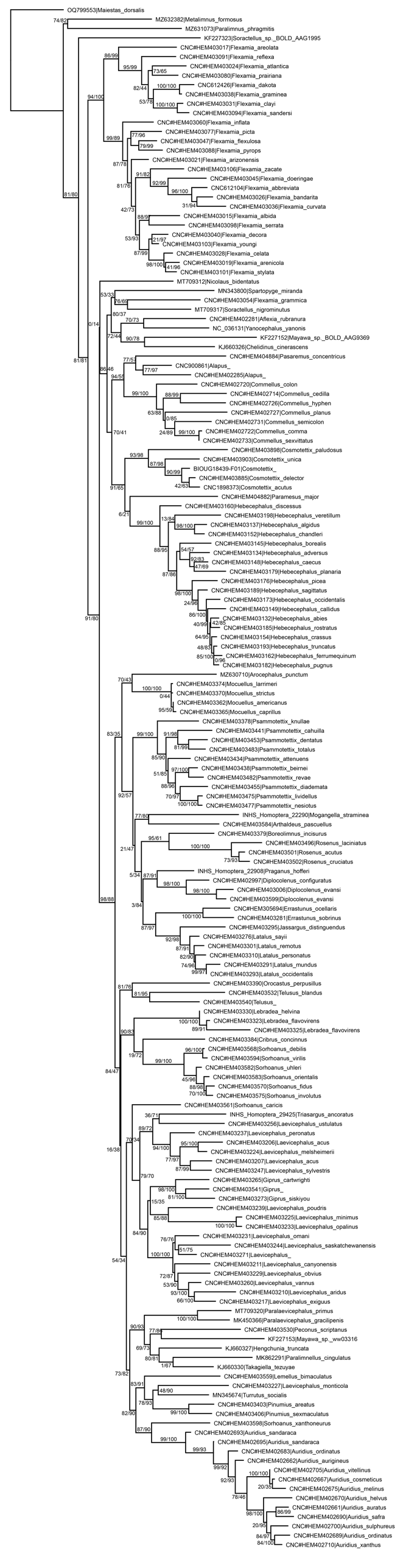

Supplement: Supplementary material 1 — Maximum likelihood tree of Paralimnini, based on 658 base pairs of cytochrome oxidase I [file zookeys-1217-273_article-126602__-s001.tif]
